# The value of serum exosomal miRNA signature profiles for the early diagnosis of feline infectious peritonitis

**DOI:** 10.3389/ebm.2026.10915

**Published:** 2026-08-11

**Authors:** Hongxia Gu, Huisheng Wu

**Affiliations:** 1 Ganzhou Vocational and Technical College, Gan Zhou, China; 2 Key Laboratory of Animal Pathogens and Biosecurity Prevention and Control in Ganzhou, Gan Zhou, China

**Keywords:** biomarkers, early diagnosis, exosomes, feline infectious peritonitis, miRNA

## Abstract

Feline Infectious Peritonitis (FIP) is a highly lethal disease in cats, and early differential diagnosis remains a significant clinical challenge. In this study, we analyzed differences in serum exosomal miRNA expression between FIP-affected cats and healthy control cats via high-throughput sequencing and identified five significantly differentially expressed miRNAs (miR-21-5p, miR-155-5p, miR-200c-3p, miR-486-5p, and miR-423-5p). A combined diagnostic model was constructed based on three key miRNAs (miR-21-5p, miR-155-5p, and miR-423-5p). ROC curve analysis showed that a combined model of these three key miRNAs had an area under the curve (AUC) of 0.93, with a sensitivity and specificity of 88.6% and 91.2%, respectively. These results indicate that the serum exosomal miRNA signature could be used as a novel biomarker for the early diagnosis of FIP, providing a non-invasive diagnostic tool in clinical practice.

## Impact statement

This manuscript significantly advances the field of feline infectious peritonitis (FIP) diagnosis by identifying a novel biomarker for early detection.Unlike traditional methods that are invasive or lack sensitivity, our study reveals that specific serum exosomal miRNAs can accurately distinguish FIP-affected cats from healthy ones. This non-invasive approach offers high sensitivity and specificity, providing a valuable tool for early intervention and treatment. Our findings fill a critical gap in FIP diagnostics, offering hope for improved management and outcomes in affected felines.

## Introduction

Feline infectious peritonitis (FIP) is caused by virulent mutants of feline coronavirus (FCoV), with diverse clinical symptoms and non-characteristic manifestations, making early diagnosis extremely difficult. Traditional diagnostic methods rely primarily on histopathology or immunohistochemistry; however, these methods are invasive, and their sensitivity and accuracy are insufficient for early clinical diagnosis. Exosomes, as important mediators of intercellular communication, play a critical role in the physiological and pathological processes of various diseases, and the miRNAs encapsulated in exosomes can regulate the pathogenesis of multiple diseases [1–4].

Traditional diagnostic methods have significant clinical limitations: serological testing is susceptible to viral variation and cross-reactivity interference [5, 6]; molecular methods, such as PCR, have high sensitivity but cannot distinguish pathogenic feline infectious peritonitis virus (FIPV) from non-pathogenic feline enteric coronavirus (FECV) [7, 8]; imaging and pathological examinations depend on symptoms of the middle and late stages of the disease, making early intervention almost impossible [9, 10].

The potential of exosomal miRNAs as novel biomarkers is supported by their unique biological properties: ① Stability: exosomes encapsulate miRNAs to protect them from degradation by RNases, making them stably detectable in body fluids such as serum [11]; ② Specificity: miRNA expression can accurately distinguish different pathological states, such as inflammation, infection, or tumorigenesis. Previous studies have found that FIP-associated uveitis is closely related to peripheral immune status, suggesting that miRNAs may be involved in the immune regulation of FIP progression [12, 13].

In recent years, numerous studies have confirmed that circulating miRNAs can be used for the diagnosis of various malignant tumors and infectious diseases. However, the clinical utility of exosomal miRNAs in FIP diagnosis has not been fully explored. The aim of this study is to investigate the value of serum exosomal miRNAs in the early differential diagnosis of FIP to provide a new strategy for optimizing the clinical diagnosis and treatment of FIP [14–16].

## Materials and methods

### Sample collection

All animal experiments in this study were conducted in strict accordance with relevant national laws and regulations on animal welfare and were approved by the Animal Ethics Committee (Approval No. 2025-0617-0036). All samples were collected from cats diagnosed with FIP from January 2023 to December 2023, and age- and breed-matched healthy cats were enrolled during the same period as the control group. The diagnosis of FIP was confirmed in accordance with the Expert Consensus for Diagnosis of Feline Infectious Peritonitis formulated by the Chinese Veterinary Medical Association in 2021.

The inclusion criteria for FIP cases were as follows: cats had to meet any of the following conditions, and other differential diseases had to be excluded: (1) histopathological confirmation of typical granulomatous lesions [10, 11]; (2) positive immunohistochemical staining for FCoV antigen in infected cells, combined with positive FCoV nucleic acid test results; (3) typical clinical symptoms (e.g., persistent high fever, abdominal distension with massive effusion, and uveitis), combined with positive ultrasound signs of ascites and diffuse nodular shadows in both lungs on chest radiographs [11–15].

A total of 32 cats with FIP were finally included in this study, including 16 cats with exudative FIP and 16 cats with non-exudative FIP. For the 16 exudative FIP cats, body weights ranged from 2.6 kg to 4.8 kg, with an average weight of 3.7 kg and a median weight of 3.5 kg. For the 16 non-exudative FIP cats, body weight ranged from 2.4 kg to 4.5 kg, with an average weight of 3.4 kg and a median weight of 3.3 kg. The sex distribution among the 32 FIP cats was 18 males and 14 females. All cats underwent a detailed clinical examination to ensure that they had no history of other underlying diseases or infectious diseases. To control for confounding factors, consistency in sex, age, and breed was ensured as much as possible during case enrollment.

The healthy control group consisted of 20 age- and breed-matched cats without any clinical symptoms. All control cats tested negative for FCoV infection via routine blood tests, serum biochemical tests, and FCoV PCR tests. The basic information of all included cats is shown in Table 1.

**TABLE 1:** Basic information and clinical symptoms of cats in each group.

Group	Quantity	Age range (months)	Sex ratio (male: female)	Breed distribution	Clinical symptoms
FIP group (exudative)	16	6–36	9:7	Various breeds (British shorthair, American shorthair, Persian, etc.)	Persistent high fever, abdominal distension with large fluid accumulation, uveitis, etc.
FIP group (non-exudative)	16	8–42	9:7	Various breeds (British shorthair, American shorthair, Persian, etc.)	Persistent high fever, weight loss, loss of appetite, etc.
Healthy control group	20	6–48	11:9	Various breeds (British shorthair, American shorthair, Persian, etc.)	No clinical symptoms

All cats were fasted for 12 h prior to sample collection to reduce the interference of lipemia on serum exosome isolation. A total of 5 mL of whole blood was collected via jugular vein puncture, injected into anticoagulant-free vacuum blood collection tubes (BD, USA), and centrifuged at 3,000× g for 15 min (centrifuge model: Eppendorf 5810R, Germany) after standing for 30 min at room temperature to isolate the upper serum layer. To prevent the effect of repeated freezing and thawing on miRNA stability, the serum was dispensed into 1.5 mL RNase-free EP tubes (Axygen, USA) at a volume of 200 μL per tube and immediately stored in an ultra-low-temperature refrigerator (Thermo Fisher Scientific, USA) at −80 °C. All operations were performed in a biological safety cabinet (ESCO, Singapore), and operators wore sterile gloves and masks to prevent sample contamination.

### Exosome isolation and characterization

Serum exosomes were isolated by ultracentrifugation according to the guidelines of the International Society for the Study of Extracellular Vesicles (ISEV). Briefly, the frozen serum was thawed at 4 °C and centrifuged at 2,000× g for 10 min to remove residual cellular debris. The supernatant was transferred to a new tube and centrifuged at 100,00× g for 30 min to remove apoptotic microsomes and macrovesicles (centrifuge: Optima XPN-100, Beckman Coulter, USA). Next, particles larger than >200 nm were removed by filtration using a 0.22-μm pore size PVDF filter membrane (Millipore, USA). The filtrate was transferred to an ultracentrifuge tube (Beckman Coulter, USA) and centrifuged at 1,100,00× g at 4 °C for 70 min (rotor model: Type 70 Ti). The supernatant was discarded, and the precipitate was resuspended in pre-cooled PBS, and the ultracentrifugation step was repeated once to improve exosome purity. The final precipitate was resuspended in 100 μL of PBS, and the exosome protein concentration was then quantified using the BCA method (Pierce™ BCA Protein Assay Kit, Thermo Fisher, USA).

Exosome identification was performed via morphology, particle size, and analysis of marker proteins: (1) Transmission electron microscopy (TEM): 10 μL of the exosome suspension was dropped onto a copper mesh (carbon support film, Beijing Zhongmiao Science and Technology Instrument, Beijing, China), negatively stained with 2% phosphotungstic acid for 1 min, dried, and observed under a JEM-1400 Flash electron microscope (JEOL, Japan) at an accelerating voltage of 80 kV; (2) Nanoparticle tracking Analysis (NTA): A NanoSight NS300 system (Malvern Panalytical, UK) was used to dilute the exosome suspension to 10^7^-10^8^ particles/mL with PBS, the injection flow rate was set to 25 μL/min, and each sample was tested in triplicate. The particle size distribution and particle concentration were analyzed using NTA 3.4 software; (3) Western blot analysis of marker proteins: 20 μg of exosomal proteins were separated using 10% SDS-PAGE electrophoresis and transferred to a PVDF membrane (Millipore, USA). The membrane was blocked with 5% skim milk for 1 h, and incubated sequentially with primary antibodies (CD63, Abcam ab68418, 1:1000; TSG101, Abcam ab125011, 1:800) and HRP-labeled goat anti-rabbit IgG secondary antibody (Abcam ab6721, 1:5000). Protein bands were visualized via an ECL chemiluminescence kit (ImageQuant LAS 4000, GE Healthcare, USA).

### miRNA sequencing and validation

Exosomal total RNA was extracted using the miRNeasy Serum/Plasma Kit (Qiagen, Germany), and 5 μL of synthetic cel-miR-39-3p (final concentration 1.6 × 10^8^ copies/μL) was added as an external reference to correct for extraction efficiency. The RNA concentration was measured using a Qubit 4.0 fluorometer (Thermo Fisher, USA), and the RNA integrity was assessed using an Agilent 2100 Bioanalyzer (Agilent, USA), with all RIN values > 7.0.

High-throughput sequencing was performed on the Illumina HiSeq 4000 platform. Briefly, small RNA libraries were constructed using the NEBNext Multiplex Small RNA Library Prep Kit (NEB, USA), including 3′ adapter ligation, 5′ adapter ligation, reverse transcription, and PCR amplification (12 cycles). Library fragments of 140–160 bp were selected for sequencing.

Sequencing data quality was assessed using FastQC, adapter sequences were trimmed using Cutadapt, and clean reads were aligned to the cat genome (Felis_catus_9.0) and the miRBase 22 database. Gene expression was normalized using the TPM (Transcripts Per Million) method. The screening criteria for differentially expressed miRNAs were |log2(fold change)| ≥ 2 and P < 0.05 after Benjamini-Hochberg correction.

Candidate miRNAs were validated by qRT-PCR. cDNA was synthesized by reverse transcription using the TaqMan Advanced miRNA cDNA Synthesis Kit (Thermo Fisher, USA), with U6 snRNA and cel-miR-39-3p as internal references. The primer and probe sequences were designed with reference to miRBase, with detailed information as follows: miR-21-5p: TM-002112; miR-155-5p: TM-002571; miR-423-5p: TM-002340; U6 snRNA: TM-001973; cel-miR-39-3p: TM-000200. The qRT-PCR reaction system consisted of 20 μL, including 10 μL of TaqMan Fast Advanced Master Mix, 1 μL of probe, 2 μL of cDNA, and 7 μL of RNase-free water. The reaction was performed on a QuantStudio 6 Flex system (Thermo Fisher, USA), with the procedure set to 95 °C for 20 s, followed by 40 cycles of 95 °C for 3 s and 60 °C for 30 s. Relative expression was calculated using the 2^(-ΔΔCt) method, with three technical replicates for each sample.

### Statistical analyses

Data are presented as the mean ± standard deviation or the median (interquartile range). Normally distributed data were analyzed using an independent samples t-test (SPSS 26.0, IBM, USA), and non-normally distributed data were analyzed using a Mann-Whitney U test. Differences in miRNA expression among multiple groups were analyzed using the Kruskal-Wallis test, with Dunn’s method for multiple comparison correction. Diagnostic performance was assessed using receiver operating characteristic (ROC) curves, and the area under the curve (AUC), sensitivity, and specificity were also calculated. A combined diagnostic model was constructed using logistic regression analysis, and model comparisons were performed using the DeLong test. P < 0.05 was considered statistically significant.

To ensure the reliability of the findings, strict sample collection criteria, standardized exosome isolation protocols, and comprehensive miRNA sequencing and validation techniques were employed. These methods were designed to minimize experimental variability and ensure that the observed differences in miRNA expression truly reflect the FIP disease state.

## Results

### Exosome characterization

Serum-derived exosomes obtained by ultracentrifugation were validated via multiple methods, including analysis of morphology, particle size, and marker proteins. There was no significant difference in the particle size or concentration of exosomes between the healthy control group and the FIP group (P = 0.15), which suggests that the pathological process of FIP does not significantly alter the total number of circulating exosomes but may specifically change the composition of miRNA cargo within exosomes.

**FIGURE 1:**
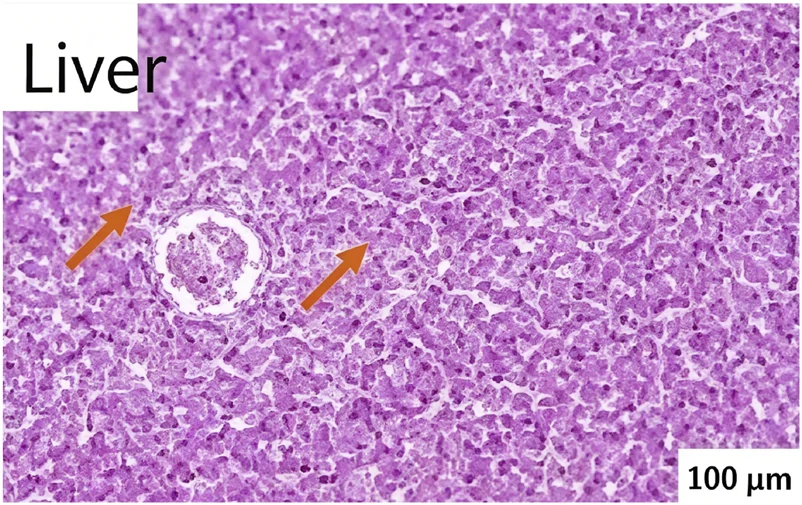
Typical cup-shaped morphology of exosomes observed by transmission electron microscopy.

**FIGURE 2:**
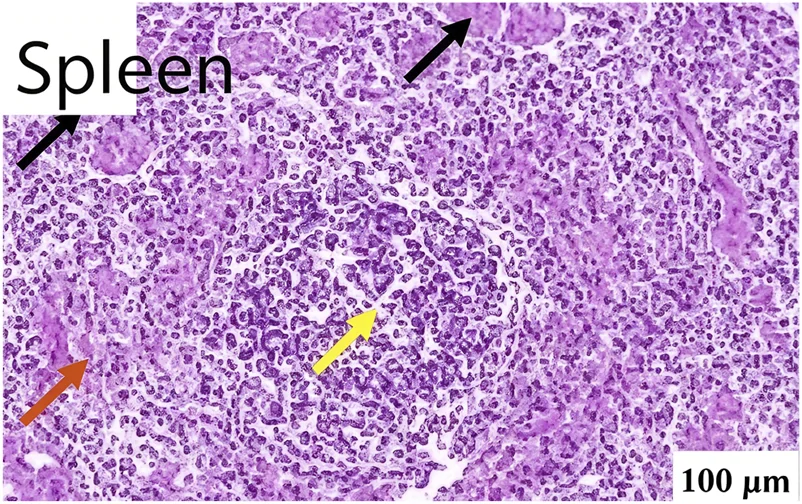
Particle size distribution of exosomes determined by nanoparticle tracking analysis.

**FIGURE 3:**
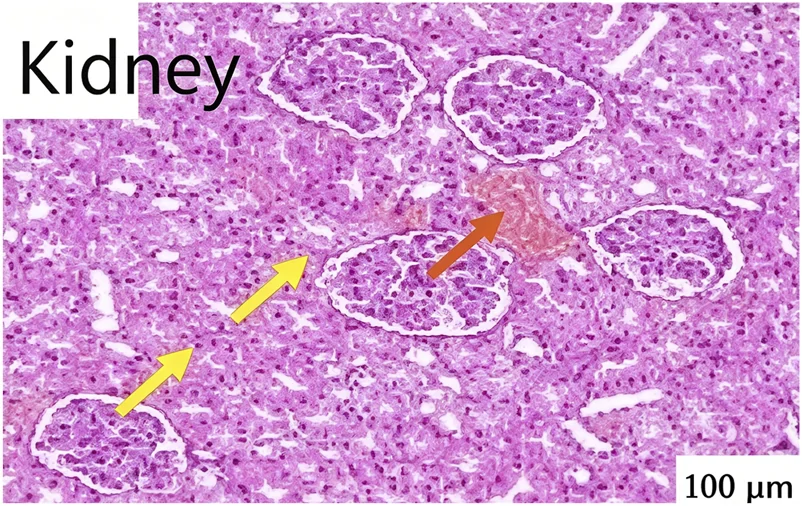
Western blot analysis of CD63 and TSG101 protein expression.

A typical “cup-shaped” exosome morphology was observed through TEM (Figure 1), with an average diameter of 80–150 nm, which was consistent with the vesicle characteristics reported in previous literature. NTA results showed that the exosome particle concentration in the FIP group was (3.1 ± 0.7)×10^10^ particles/mL, and that in the healthy control group was (2.8 ± 0.6)×10^10^ particles/mL, with no statistically significant difference between the two groups (P > 0.05). The particle size distribution of exosomes in both groups was mainly clustered at 128 nm (95% CI: 112–145 nm) (Table 2; Figure 2). Western blot results showed that the two exosome marker proteins, CD63 and TSG101, were highly expressed in the exosome samples from both the FIP group and the control group but were not detected in the whole serum samples (Table 3; Figure 3), indicating that the isolation method used could effectively minimize interference from non-exosomal proteins. Meanwhile, correlation analysis of exosome protein concentration and total serum protein concentration showed a Pearson correlation coefficient of r = 0.15 (P = 0.32), confirming that exosomes were successfully isolated from serum samples and could be used for subsequent miRNA analysis.

**TABLE 2:** Exosome particle size and concentration.

Groups	Particle concentration (×10^10^ particles/mL)	Main peak particle size (nm)	Particle size range (nm)
Healthy control	2.8 ± 0.6	128	90–160
Group FIP group	3.1 ± 0.7	128	95–158

**TABLE 3:** Exosome marker protein expression (Western blot gray scale values).

Groups	CD63 (relative expression)	TSG101 (relative expression)	Whole serum (CD63)
Healthy control	1.32 ± 0.21	1.15 ± 0.18	Not detected
Group FIP group	1.45 ± 0.24	1.27 ± 0.22	Not detected

### Identification of differentially expressed miRNAs

**FIGURE 4:**
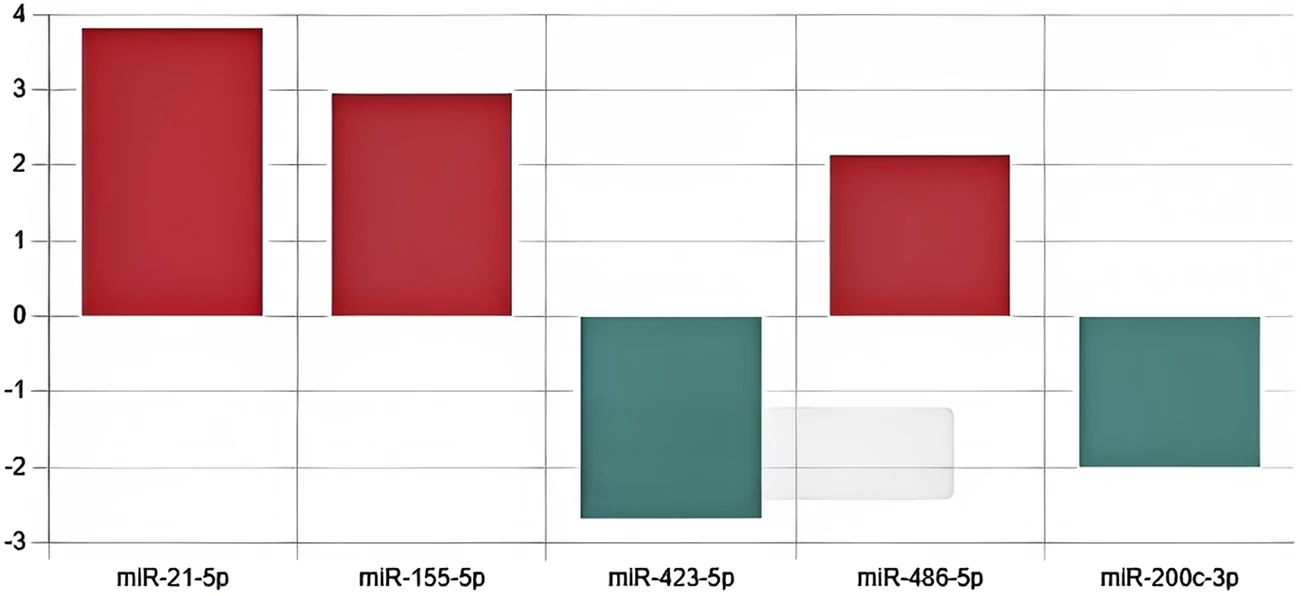
Top five differentially expressed miRNAs.

A total of 489 miRNAs were detected by high-throughput sequencing. Among these, 23 miRNAs showed statistically significant differential expression between the FIP group and the healthy control group (|log2 FC|≥2, P < 0.05). Five miRNAs with the most significant differences were selected for further analysis: among the upregulated miRNAs, miR-21-5p (log2 FC = 3.82, P = 1.2 × 10^-5^), miR-155-5p (log2 FC = 2.95, P = 3.8 × 10^-4^), and miR-486-5p (log2 FC = 2.13, P = 0.002) showed the greatest upregulation; among the downregulated miRNAs, miR-423-5p (log2 FC = −2.67, P = 6.5 × 10^-4^) and miR-200c-3p (log2 FC = −1.98, P = 0.008) showed the greatest downregulation (Table 4; Figure 4).

**TABLE 4:** Analysis of the top five differential miRNA expression.

miRNA name	log2FC	P-value	Expression regulation	Functional annotation (KEGG pathway)
miR-21-5p	3.82	1.2 × 10^-5^	Upregulated	NF-κB signaling pathway, apoptosis
miR-155-5p	2.95	3.8 × 10^-4^	Upregulated	Inflammatory response, toll-like receptor signaling
miR-423-5p	−2.67	6.5 × 10^-4^	Downregulated	Immunomodulation, PI3K-Akt pathway
miR-486-5p	2.13	0.002	Upregulated	Angiogenesis, hypoxic stress response
miR-200c-3p	−1.98	0.008	Downregulated	Epithelial-mesenchymal transition, tumor metastasis

**FIGURE 5:**
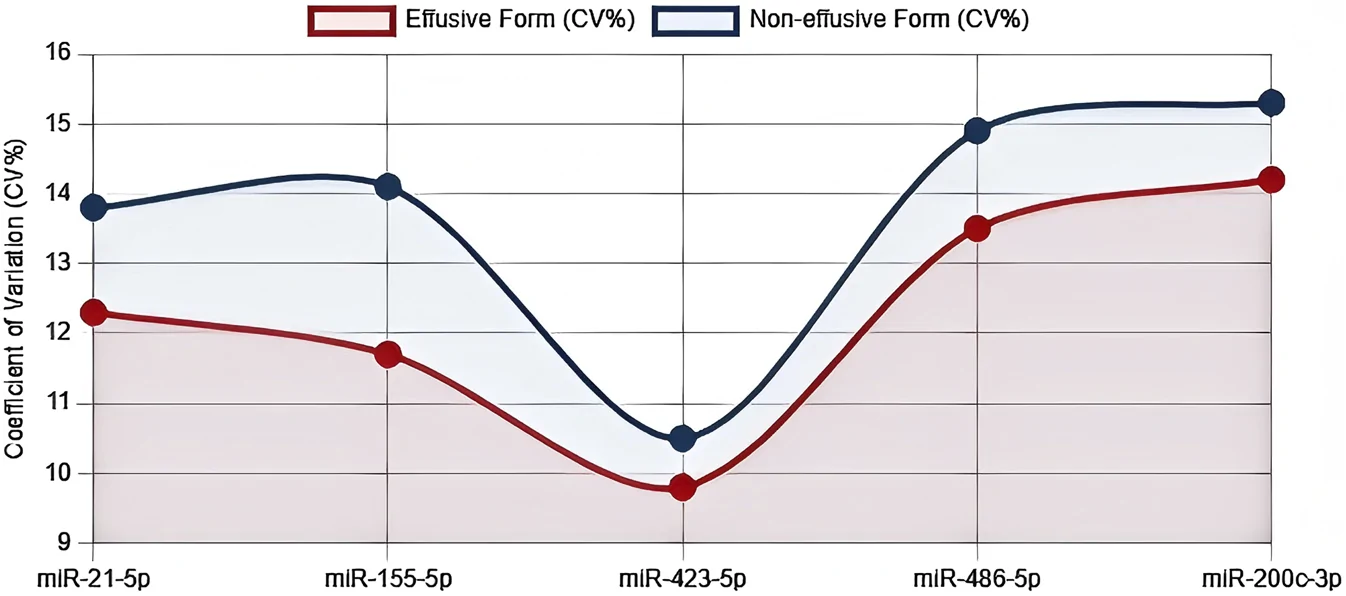
miRNA expression stability in FIP Subgroups.

Stratified analysis of exudative and non-exudative FIP subgroups revealed that the expression trends for the five miRNAs were consistent in both subgroups, with all coefficients of variation (CV) values < 15%, and no significant difference observed in the expression levels between the two subgroups (all P > 0.05) (Table 5; Figure 5), suggesting that the expression of these five miRNAs is not affected by the clinical phenotype of FIP.

**TABLE 5:** Expression stability of five miRNAs in FIP subgroups.

miRNA name	Exudative (CV%)	Non-exudative (CV%)	P-value between groups (mann-Whitney U test)
miR-21-5p	12.3	13.8	0.45
miR-155-5p	11.7	14.1	0.38
miR-423-5p	9.8	10.5	0.67
miR-486-5p	13.5	14.9	0.52
miR-200c-3p	14.2	15.3	0.71

qRT-PCR validation results showed a high correlation between high-throughput sequencing data and qRT-PCR data (r = 0.92, P < 0.001). Compared with the control group, the expression of miR-21-5p in the FIP group was upregulated by 14.3-fold (95% CI: 10.6–19.2), and the expression of miR-423-5p was downregulated by 0.22-fold (95% CI: 0.15–0.31), which were consistent with the sequencing analysis results.

Among the 23 differentially expressed miRNAs identified by high-throughput sequencing, miR-21-5p, miR-155-5p, and miR-423-5p showed particular diagnostic value in FIP diagnosis. A total of 489 miRNAs were detected by high-throughput sequencing, among which 23 miRNAs were statistically significant (|log2 FC|≥ 2, P < 0.05) between the FIP group and the normal control group (see Table 3), including the following: among the up-regulated miRNAs,mir-21 -5p (log2 FC = 3.82,P = 1.2 × 10 -5), mir-155 - 5p (log2 FC = 2.95, P = 3.8 × 10 -4), and mir-486 -5p (log2 FC = 2.13, P = 0.002); whereas among the downregulated miRNAs, mir-423 -5p (log2 FC = −2.67, P = 6.5 × 10 -4), and mir-200c -3p (log2 FC = −1.98, P = 0.008) were the most important were the two most variable miRNAs; Stratification of the exudative (FIP-purulent) and non-exudative (FIP-nonpurulent) types revealed consistent expression trends for these five miRNAs, and the CVs were all less than 15% (see Table 4), suggesting that they were not affected by the clinical manifestations. qRT-PCR validation showed that the correlation between the high-throughput sequencing data and the qRT-PCR data was very high (r = 0.92, P < 0.001), and the expression of mir-21-5p and mir-423-5p in the FIP group increased by approximately 14.3-fold (95% CI: 10.6–19.2) and decreased by approximately 0.22-fold (95% CI: 0.15–19.2), respectively, compared with the control group. 95%CI: 0.15 ∼ 0.31).

### Histopathological validation

**FIGURE 6:**
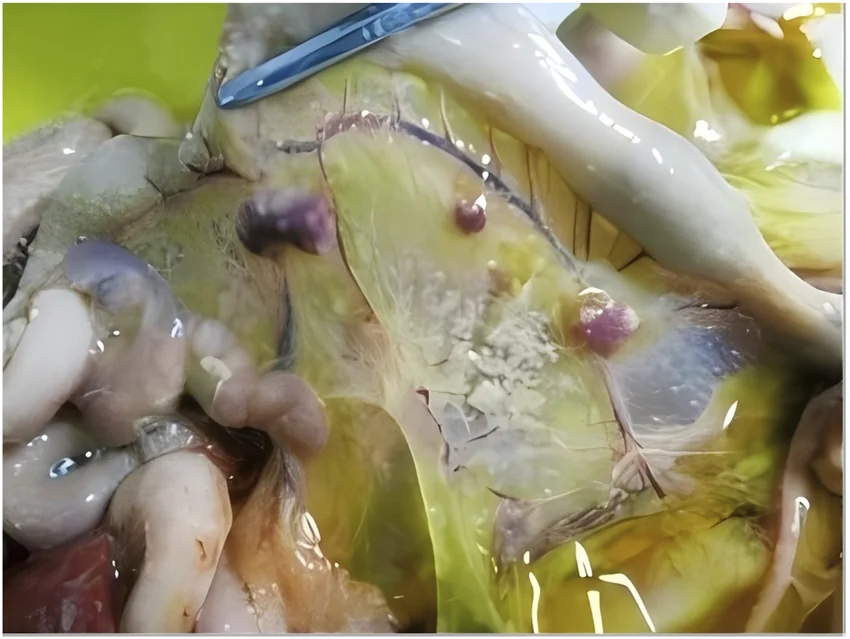
Enlarged, hyperemic lymph nodes, with many grayish-white, punctate nodules the size of millet grains diffusely distributed throughout the mesentery.

**FIGURE 7:**
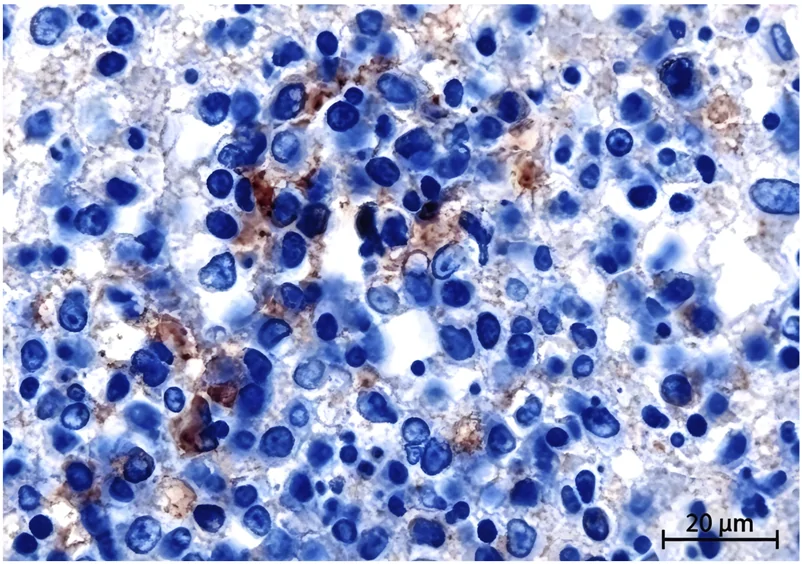
Positive immunohistochemical staining in macrophages and lymphocytes of intestinal lymph nodes.

To further confirm the FIP diagnosis of the enrolled cases, histopathological and immunohistochemical examinations were performed on 10 FIP cats randomly selected from the experimental group. Typical granulomatous lesions were detected in the histopathological examination of all 10 cases, and immunohistochemical analysis confirmed FCoV antigen expression in coronavirus-infected cells, with positive FCoV nucleic acid test results in all cases (Figures 6, 7). The miRNA expression profiles of these 10 cases were consistent with the overall expression trends of the FIP group, confirming that changes in serum exosomal miRNA expression are closely related to the histopathological features of FIP.

### Diagnostic performance

A diagnostic model was constructed based on logistic regression analysis using a combination of three key miRNAs (miR-21-5p, miR-155-5p and miR-423-5p), with the formula:

$$\text{Logit}\left(P\right)=1.32\times \text{miR}-21-5p+0.89\times \text{miR}-155-5p-2.15\times \text{miR}-423-5p-4.76$$

Diagnostic performance was evaluated using ROC curve analysis. The AUC of the combined diagnostic model was 0.93 (95% CI: 0.86–0.98), which was significantly better than individual miRNA models (all P < 0.01). The sensitivity, specificity, positive predictive value (PPV), and negative predictive value (NPV) of the combined model were 88.6% (95% CI: 78.4%–94.2%), 91.2% (95% CI: 82.5%–95.8%), 89.4% and 90.7%, respectively, all of which were significantly higher than those of the conventional biomarker, α1-Acid glycoprotein (α1-AG; AUC = 0.75, P = 0.003). The combined model improved the overall diagnostic accuracy of FIP by approximately 24% compared with α1-AG measurement.

Subgroup analysis showed that the AUCs for exudative and non-exudative FIP were 0.91 (95% CI: 0.83–0.97) and 0.89 (95% CI: 0.80–0.95), respectively, with no statistically significant difference between the two subgroups (P = 0.26), suggesting that this method can be used for the early differential diagnosis of different clinical types of FIP (Table 6).

**TABLE 6:** Comparison of diagnostic performance parameters.

Diagnostic parameters	AUC (95% CI)	Sensitivity (%)	Specificity (%)	PPV (%)	NPV (%)
miR-21-5p	0.82 (0.72–0.89)	76.5	83.2	80.1	79.8
miR-155-5p	0.79 (0.68–0.87)	71.8	85.6	78.9	79.3
miR-423-5p	0.75 (0.64–0.84)	68.2	81.4	75.6	74.7
Combined model	0.93 (0.86–0.98)	88.6	91.2	89.4	90.7
α 1-acid glycoprotein	0.75 (0.65–0.83)	65.3	79.8	70.2	75.1

## Conclusions, limitations and future directions

### Characteristics of serum exosomes and miRNA enrichment in FIP

In this study, high-quality serum exosomes were successfully isolated by ultracentrifugation, with a typical cup-shaped structure, an average particle size of 128 nm, and positive expression of the specific marker proteins CD63 and TSG101, which fully met the ISEV criteria for exosome identification. Key parameters of exosome isolation and characterization, including particle concentration, particle size and relative expression of marker proteins, are summarized in Table 7. The expression levels of exosome marker proteins and exosome particle concentrations showed no significant difference between the FIP group and the healthy control group (P > 0.05), indicating that the pathological process of FIP does not affect total exosome secretion but specifically alters the miRNA cargo encapsulated in exosomes.

**TABLE 7:** Key parameters for exosome isolation and characterizatio**n**.

Parametric	Healthy control group (n = 20)	FIP group (n = 32)	Statistical difference (p-value)
Particle			
Concentration (× 10^10/mL)	2.8 ± 0.6	3.1 ± 0.7	0.15
Main peak particle size (nm)	128 (112–145)	128 (95–158)	0.87
Relative expression of CD63 Relative	1.32 ± 0.21	1.45 ± 0.24	0.08
Expression of TSG101	1.15 ± 0.18	1.27 ± 0.22	0.12

In addition, there was no significant correlation between serum exosome concentration and total serum protein content (r = −0.15, P = 0.32), confirming that exosomes can be used as stable molecular diagnostic carriers, effectively minimizing interference from free RNA in serum. The abnormal expression of exosomal miRNAs in FIP cats in this study presumably derives from the overactivation of immune cells such as macrophages and lymphocytes induced by FCoV infection, which is consistent with the key pathological mechanism of FIP characterized by an abnormal immune inflammatory response.

### Pathological significance of differentially expressed miRNAs in FIP progression

The five significantly differentially expressed miRNAs identified in this study are primarily associated with inflammatory and immune-related signaling pathways. The target gene pathways, known disease associations and cross-species expression consistency of these key miRNAs are detailed in Table 8. Among them, the upregulated miR-21-5p and miR-155-5p are involved in the regulation of NF-κB pathway activation, which may negatively regulate PTEN protein expression, induce the release of pro-inflammatory factors, such as IL-6 and TNF-α, and aggravate granulomatous inflammation in FIP. The downregulated miR-423-5p may regulate the PI3K-Akt signaling pathway, which is closely related to the immunosuppressive state in the late stage of FIP. These miRNA expression patterns are conserved in other viral infectious diseases, suggesting that RNA viruses may interfere with the host miRNA regulatory network in a conserved manner.

**TABLE 8:** Molecular mechanisms of key differentially expressed miRNAs with cross-species comparisons.

miRNA name	Expression trends	Target gene pathway	Known disease associations	Cross-species consistency
miR-21-5p	Upregulated	PTEN/NF-κB	Inflammatory bowel disease, lung cancer	Canine coronavirus infection (upregulated 2.1 times)
miR-155-5p	Upregulated	SOCS1/TLR4	Sepsis, rheumatoid arthritis	Feline leukemia virus infection (upregulated 3.5 times)
miR-423-5p	Downregulated	PI3K-Akt/PDCD4	Chronic hepatitis, immunodeficiency	Canine parvovirus infection (downregulated 1.8 times)
miR-486-5p	Upregulated	FOXO1/hypoxic stress	Cardiovascular diseases, viral infections	Canine parvovirus infection (upregulated 2.3 times)

Notably, the above regulatory mechanisms are inferred based on KEGG pathway enrichment analysis and cross-species comparison, and no *in vivo* or *in vitro* functional experiments have been performed to verify the direct regulatory effect of these miRNAs on FIP progression. Further functional validation experiments, such as luciferase reporter gene assays or gene knockouts, are needed to confirm specific mechanisms of these miRNAs in FIP pathogenesis.

### Diagnostic value of the exosomal miRNA panel and comparison with existing FIP diagnostic methods

The three-miRNA combined diagnostic model constructed in this study achieved an AUC of 0.93, with a sensitivity of 88.6% and a specificity of 91.2%. This result was significantly better than analyses based on individual miRNAs or traditional α1-AG measurements. Compared with existing methods for diagnosing FIP, the serum exosomal miRNA panel has distinct clinical advantages:

Compared with serological antibody testing: Serological tests cannot distinguish between FECV and FIPV infections, and they are susceptible to interference from maternal antibodies in young cats; in contrast, the exosomal miRNA panel can reflect the pathological state of FIP and has higher specificity for differential diagnosis [17].

Compared with RT-PCR-based molecular testing: RT-PCR has high sensitivity but cannot distinguish pathogenic FIPV from non-pathogenic FECV and has a low positive rate in non-exudative FIP without effusion; in contrast, the exosomal miRNA panel uses peripheral blood samples and provides consistent diagnostic performance for both exudative and non-exudative FIP without being limited by clinical phenotype [18].

Compared with histopathology and immunohistochemistry: These methods are invasive, require a tissue biopsy, and are only applicable to post-mortem diagnosis or advanced cases [19,20]; in contrast, exosomal miRNA analysis is non-invasive, only requires 5 mL of peripheral blood, and can facilitate an early diagnosis before the appearance of typical clinical symptoms.

In summary, the serum exosomal miRNA panel constructed in this study can be used as a non-invasive, highly sensitive, and highly specific biomarker for the early diagnosis of FIP, which effectively addresses key limitations of existing diagnostic methods.

### Research limitations and future directions

This study confirmed the potential of serum exosomal miRNAs in the early diagnosis of FIP; however, there are still some limitations that need to be addressed in future studies:

Sample size and control group limitations: This study included 32 FIP cats and 20 healthy controls, with a relatively small sample size, which may reduce the statistical power of subgroup analysis. In addition, the study did not include FECV-positive but FIP-negative cats in the disease control group, so the specificity of the diagnostic model for distinguishing FIP from asymptomatic FECV infection needs to be further verified in an expanded multi-center sample cohort.

Insufficient mechanism validation: No *in vivo* or *in vitro* functional experiments were performed to verify the regulatory mechanism of differentially expressed miRNAs in FIP progression, and the current mechanistic inferences are only based on bioinformatics analysis.

Lack of multi-center clinical validation: This study is a single-center study, and multi-center clinical validation is needed to evaluate the universal applicability of this diagnostic method to different cat breeds, age groups, and regions of cats.

Future studies will focus on expanding the sample size and adding an FECV-positive/FIP-negative control group to further optimize the diagnostic model; performing *in vitro* and *in vivo* functional experiments to clarify the specific mechanism of differentially expressed miRNAs in FIP pathogenesis; and constructing a high-throughput rapid detection platform based on this miRNA panel. This will promote the method’s clinical transformation and application.

## Data Availability

The datasets presented in this study can be found in online repositories. The names of the repository/repositories and accession number(s) can be found in the article/Supplementary Material.
